# Special Issue: Psychosocial Considerations for Children and Adolescents Living with a Rare Disease

**DOI:** 10.3390/children9071099

**Published:** 2022-07-21

**Authors:** Maureen E. Lyon, Lori Wiener

**Affiliations:** 1 Children’s National Hospital, Center for Translational Research, Washington, DC 20010, USA; 2 School of Medicine and Health Sciences, George Washington University, Washington, DC 20052, USA; 3 Pediatric Oncology Branch, National Cancer Institute, National Institutes of Health, Bethesda, MD 20892, USA; wienerl@mail.nih.gov

## 1. Introduction

This Special Issue of the journal *Children* constitutes an opportune moment to reflect on the psychosocial needs of children living with rare diseases and of their families. As medical advances, treatments, and developments have enabled many of these children to survive infancy and to live into adulthood, progress brings with it concerns and opportunities to enhance the psychosocial quality of life of children living with rare diseases, and of their families.

In August of 2021, we released a call for papers whereby healthcare providers could share their experience and research on the psychosocial needs of children living with rare diseases, and the needs of their families. Our call resulted in 13 accepted peer-reviewed submissions. The manuscripts covered a diverse range of topics and contributions from around the globe, including Asia (Taiwan), Australia, Europe (Germany, Italy, Sweden, and The Netherlands) and the United States.

We acknowledge that, as is often the case with Special Issues and their time constraints, the manuscripts within this Special Issue do not cover or represent all the potentially important contributions to the topic. However, as international perspectives are shared, we hope this Special Issue leads to future research collaborations. It is also our hope that the data presented in this Special Issue will ultimately reduce the systemic and structural inequities that place children with rare diseases at unfair, unjust, and avoidable disadvantages with respect to their quality of life and that of their families.

This Special Issue reflects the current state of psychosocial research, which is primarily qualitative in nature. There are no scientifically rigorous randomized clinical trials to create an evidence base of effective psychosocial interventions for the provision of care to children with rare diseases and to their families; nevertheless, the papers within this Special Issue provide a reflection on the state of the science, including ideas about future research and practice. In this next section we share observations about the contributions made by each of the 13 articles, which cover a diverse range of topics.

## 2. Contributions to the Special Issue

Belzer, Wright, Goodwin, Singh, and Carter provide a thorough, thoughtful, and comprehensive overview of psychosocial considerations for the child with a rare disease, including recommendations and a call to action [1]. Of particular importance, the authors note the experience of stigma and social isolation amidst the medicalization of homes and family lives, and the need for care coordination. The authors call for a focus on the intersectionality of identities (e.g., gender, race, and poverty), experiences, and care models. The impact of the social determinants of health, known to contribute to inequity outcomes, has not been fully characterized; this is, in part, because of small sample sizes. The importance of the child voice (when possible), of the family as part of the care and research team, of gender differences in caregiving, of sibling caregiving, of access barriers in the community, of electronic health record functionality with documentation, and of the transition to adult healthcare is also reviewed here and further explicated in the articles within this Special Issue.

Each rare disease is unique with respect to the specific medical and psychosocial needs associated with the condition. Additionally, many studies only include the parent perspective because the child’s rare disease involves communication and/or neurocognitive disorders that preclude the child’s participation. Sharping and colleagues identify the unmet needs of the parents of children with urea-cycle disorders residing in Germany [2]. Using the validated Parental Need Scale for Rare Diseases Questionnaire, 59 parents reported on the needs of 24 children. Close to half of the parents reported a need for information on available services, and one-third on the need for additional information on the development of their children. More than two-thirds reported a need for additional support such as support groups or psychological counseling. The authors conclude that the findings underscore the importance of family-centered approaches to care. We concur that the template they used for their assessment of family burden could be used for children with other rare diseases, increasing the replicability of study findings, as children with rare diseases and their families share many commonalities in their need for knowledge about their or their children’s conditions and desire for support.

Similarly, through data collected via a structured psychosocial interview and the Distress Thermometer/Problem Checklist, Lockridge and colleagues [3] found that patients (*n* = 63) with Multiple Endocrine Neoplasia 2 (MEN2B) and Medullary Thyroid Carcinoma (MTC) identified the need for information about available services and education about MTC as high-priority. MEN2 is a genetic cancer syndrome for which there are limited data pertaining to the quality of life and psychosocial experiences of persons affected. Over half of the pediatric patients reported experiencing attention challenges and difficulty concentrating. While pediatric and adult patients identified pain as interfering with their mood and daily activities, the parents of pediatric patients reported mood shifts as most concerning, thus highlighting the importance of both the child voice and parental perspectives. The children and parents agreed that they would want to meet others with this rare condition. The study suggests that the psychological impact of living with MEN2 and MTC extends beyond changes in physical attributes, daily life limitations, and pain and; therefore, it speaks to opportunities for educational and mental-health intervention and further research.

Beckwith-Wiedemann syndrome (BWS) is a rare overgrowth disease and is not usually associated with intellectual delay. In a cross-sectional exploratory study in Italy, assessing psychosocial difficulties in preschool-age children with BWS, Butti and colleagues [4] administered two standardized questionnaires to 30 parents—the Child Development Inventory and the Child Behavior Checklist. The authors found that overall, BWS was not associated with specific behavioral problems; however, at the individual level, almost a quarter of the sample had scores in the borderline range on the withdrawal scale, and half had scores within in the borderline or critical range in the social domain. Increasing age was associated with higher behavioral and developmental difficulties. Social withdrawal problems were independent of developmental difficulties in the social domains. The authors speculate that children with BWS might become more aware of their condition as they begin spending more time with their peers in social contexts outside the family. They recommend that children with BWS receive routine psychosocial assessment of emotional and psychosocial development as they enter kindergarten and elementary school. This could have beneficial effects on the national health system in Italy, reducing costs associated with the long-term consequences of neglected emotional–behavioral problems.

In a mixed-methods study, Chu and colleagues [5] explored gender differences in parenting stress, health outcomes, and illness perceptions among 100 family caregivers (42 men and 58 women) caring for children with genetic or rare diseases in Taiwan. Measures included the Pediatric Inventory for Parents (PIP) to assess caregiver distress, the Center for Epidemiological Studies Depression Scale Short Form (CES-D Sort Form) to assess caregiver depression, and the Satisfaction with Life Scale (SWLS) to assess life satisfaction [5]. Open-ended questions were theoretically informed, using Leventhal’s Common-Sense Model of Illness Representation. Consistent with prior research, most female caregivers served as the primary caregiver and provided more caregiving, while experiencing high levels of parenting distress and depressive symptoms compared with male caregivers. The authors identified a gender discrepancy in illness perception (negative consequences requiring disease control vs. quality of life), which may have contributed to the higher levels of stress and depressive symptoms in female caregivers than in males.

The standardized documentation of psychosocial concerns is the first step in improving the ability of healthcare providers to identify and intervene in psychosocial concerns and their risk factors. The documentation of psychosocial distress and its antecedents in children with rare diseases is often not captured in the medical record, as illustrated in McCarthy and colleagues’ article [6]. The medical records of patients with rare or life-limiting chronic conditions (*n* = 60) being followed by a pediatric complex care coordination program in the United States were reviewed. The authors extracted both structured data elements and narrative text from the most recent visit with the clinician. Topics related to psychosocial distress were documented in notes, including child and parent emotional problems, parent social support, sibling emotional or physical problems, family structure, and financial concerns. However, 35% of the notes lacked any mention of psychosocial concerns and mention of parents’ emotional health or concerns was largely absent. Risk factors and vulnerabilities of the family system (i.e., financial, sibling) were also rarely captured. The authors emphasized the need for universal psychosocial screening using structured, evidence-based tools, systematically entered into the medical record as a way to contribute to an integrated medical and behavioral service model.

Among nonhuman primates, siblings are “helpers at the nest.” Therefore, it is not surprising that the siblings of children with rare diseases also function in this role, as demonstrated in the studies of Wawrzynski and colleagues [7] in the United States and Kreicbergs and colleagues in Sweden [8]. Their studies highlight the need for social support for the siblings as well as the patients. In semi-structured interviews of siblings aged 12–17 years, Wawrzynski and colleagues constructed ecomaps of support networks, including types of support and of support provider. Support networks ranged from 2–10 individuals, with mothers, fathers, close friends, and siblings, with and without cancer being major supports, in that order. We concur that this foundational knowledge of sibling networks will contribute to the design of interventions to improve support for the siblings of children with rare diseases, including cancer.

Little is known about the need for information and the involvement of the siblings of children with palliative care needs. Kreicbergs and colleagues, using four standardized communication tools (See–Hear–Do pictures, including the empty body as a separate element, Bear cards, and words originating from previous sibling research), conducted a conventional content analysis of the responses of nine siblings aged 6–14 years [8]. Most striking was that these siblings expressed an awareness that their brother or sister would die—”not if, but when”. School was perceived as a place for leisure, friends, and learning. Relentless feelings of guilt and self-blame, as well as themes of loss and separation were elicited. Nevertheless, these siblings also felt they were part of a special, happy family.

Several other papers address palliative care needs for children and adolescents living with rare conditions. Aoun and colleagues assessed the support needs of 28 parents whose children were receiving pediatric palliative care (*n* = 20 with non-cancer diagnosis, *n* = 8 with cancer diagnosis) [9]. With the study conducted in Australia, the authors used structured telephone interviews upon parents’ completion of an intervention using the Carer Support Needs Assessment Tool (CSNAT), a process for assessing the palliative care needs of children and their families. The interviews were audio-recorded and transcribed verbatim. The authors found that the parents appreciated a systematic approach in engaging them in conversations about both their needs and solutions to address them. Similar to other studies, the interviews elicited the following themes: caregiving challenges; perceived gaps in psychosocial care and feelings of isolation; and validation and empowerment when participating in the CSNAT intervention, which helped them identify strategies and receive support in response to their needs. Nevertheless, parents were left wanting practical psychosocial and emotional support. Aoun and colleagues recommend that palliative care services build stronger partnerships with supportive community networks through compassionate community volunteer models of care to address the non-clinical needs of families whose children are receiving end-of-life palliative care.

The question of whether or not pediatric advance care planning (pACP) matters to the parents of children with rare diseases, particularly for those children who are unable to participate in decision making, is only beginning to be explored. Fratantoni and colleagues beta tested a pACP intervention with six families [10]. Their article describes a qualitative analysis of structured interviews examining what parents thought mattered most to their child and what they would want their doctor to know. Five themes emerged that might guide future interventions: getting out and moving freely; feeling included and engaged; managing symptoms and disease burden; coordinating care among the many care team members; and managing today and planning for the future. The parents strived to be effective advocates on their children’s behalf.

Brunetta and colleagues conducted a systematic review of the literature on pACP, with a focus on how to operationally define age-appropriate pACP for children living with a life-limiting condition [11]. They identified 18 unique tools. These tools primarily assessed the preferences of the children and their families concerning their goals for care and end-of-life treatment preferences. In most studies, the children were adolescents who were able to participate in decision making. This article is well-organized, beginning with evidence from randomized control trials, observational studies, mixed-methods studies, qualitative studies, and descriptive studies. The authors identify six factors influencing age-appropriate care from the literature: willingness to participate; decision-making capacity; a child’s understanding of their own medical process; cognitive impairment; the development of a social identity (defined as an awareness of self and others that influences children’s preferences and goals in pACP); and legal responsibilities. The authors call for a more explicit explanation for the choice of age. For example, in adapting adult models for adolescents, it is important to address how the adaptations meet the developmental needs or capacities of the children studied. The authors also call for future studies to specify race and ethnicity.

Two studies addressed the needs of adolescents and young adults living with a chronic or rare condition. A quality-improvement study assessing the needs of adolescents and young adults (*n* = 89) with neurofibromatosis type 1, cancer, primary immunodeficiencies, or sickle cell disease, and of their caregivers (*n* = 37), was conducted by Allen and colleagues [12]. The subjects completed a survey developed for this study to identify a range of informational and service-related needs. Consistent with the other studies in this Special Issue, there was an overwhelming desire for information about their specific disease. The authors conclude that this is a critical and largely unmet component of care which requires the development and implementation of targeted educational and psychosocial interventions. Considering most adolescents and young adults have access to smartphone apps and web-based services, the authors suggest that future research should utilize digital technologies to expand services and address informational needs.

The transition to adulthood for youth living with chronic illnesses is complex. Sandquist and Lyon—in partnership with Davenport and Monaco, who are parents of children with rare diseases—provide a useful international review of the literature on challenges specific to the transition to adulthood for youth living with rare diseases [13]. Their review found that transitional support is lacking, particularly for maturing psychosocial needs. Many programs that do exist assume the young person can participate in decision making and live independently, which may not be the case for many young people with rare diseases. The parents of children with neurological conditions that impair decision making and/or independent living are often surprised to discover that they need to establish legal guardianship over their children when their child becomes a legal adult and prove their competency as caregivers to the government. The barriers and challenges to transition to adult care are identified, including the need for programmatic support. The authors conclude that a large portion of children with rare diseases are underserved and experience health disparities in the transition.

While the papers published in this Special Issue provide important new knowledge, more work is required in several areas. Discovering effective approaches to improving the quality of life of children with rare diseases and of their families necessitates addressing the social determinants of health, which, in turn, should inform clinical practice and policy. We need to examine the systemic and structural problems that contribute to health disparities and consider ascertaining psychosocial needs through new systems and models of care. Macro-level interventions at the population or community-health level can help meet the psychosocial challenges that persistently affect children living with rare diseases and their families. Finally, we recognize that, along with the caveats of what is still needed, there are some wonderful programs available for children, adolescents, and young adults living with rare conditions. Unfortunately, it is not possible to list them all here.
